# Utilizing hydrothermal time models to assess the effects of temperature and osmotic stress on maize (*Zea mays* L.) germination and physiological responses

**DOI:** 10.1186/s12870-023-04429-y

**Published:** 2023-09-07

**Authors:** Waqif Khan, Sumbal Shah, Abd Ullah, Sami Ullah, Fazal Amin, Babar Iqbal, Naveed Ahmad, Mostafa A. Abdel-Maksoud, Mohammed k. Okla, Mohamed El-Zaidy, Wahidah H. Al-Qahtani, Shah Fahad

**Affiliations:** 1 Institute of Grassland Science, Northeast Normal University, Key Laboratory of Vegetation Ecology, Ministry of Education, Changchun, China; 2 Department of Botany, University of Peshawar, Peshawar, 25120 Pakistan; 3 Xinjiang Key Desert Plant Roots Ecology and Vegetation Restoration Laboratory, Xinjiang Institute of Ecology and Geography, Chinese Academy of Sciences, 818 South Beijing Road, Urumqi, 830000 Xinjiang China; 4 State Key Laboratory of Desert and Oasis Ecology, Xinjiang Institute of Ecology and Geography, Chinese Academy of Sciences, Urumqi, 830000 Xinjiang China; 5 School of Environment and Safety Engineering, Jiangsu University, Zhenjiang, 212000 China; 6 Joint Center for Single Cell Biology, Shanghai Collaborative Innovation Center of Agri-Seeds, School of Agriculture and Biology, Shanghai Jiao Tong University, Shanghai, 200240 China; 7 Botany and Microbiology Department, College of Science, King Saud University, P.O. Box 2455, 11451 Riyadh, Saudi Arabia; 8 Department of Food Sciences & Nutrition, College of Food and Agricultural Sciences, King Saud University, P.O. Box 270677, 11352 Riyadh, Saudi Arabia; 9 Department of Agronomy, Abdul Wali Khan University Mardan, Mardan, 23200 Khyber Pakhtunkhwa Pakistan

**Keywords:** Abiotic stress, Antioxidant mechanism, Germination, Hydrothermal time model, Physiological responses

## Abstract

The application of germination models in economic crop management makes them extremely useful for predicting seed germination. Hence, we examined the effect of varying water potentials (Ψs; 0. − 0.3, − 0.6, − 0.9, − 1.2 MPa) and temperatures (Ts; 20, 25, 30, 35, 40 °C) on maize germination and enzymatic antioxidant mechanism. We observed that varying Ts and Ψs significantly influenced germination percentage (GP) and germination rate (GR), and other germination parameters, including germination rate index (GRI), germination index (GI), mean germination index (MGI), mean germination time (MGT), coefficient of the velocity of germination (CVG), and germination energy (GE) (*p* ≤ 0.01). Maximum (87.60) and minimum (55.20) hydro-time constant (θH) were reported at 35 °C and 20 °C, respectively. In addition, base water potential at 50 percentiles was highest at 30 °C (15.84 MPa) and lowest at 20 °C (15.46 MPa). Furthermore, the optimal, low, and ceiling T (To, Tb and Tc, respectively) were determined as 30 °C, 20 °C and 40 °C, respectively. The highest θT1 and θT2 were reported at 40 °C (0 MPa) and 20 °C (− 0.9 MPa), respectively. HTT has a higher value (R2 = 0.43 at 40 °C) at sub-optimal than supra-optimal temperatures (R2 = 0.41 at 40 °C). Antioxidant enzymes, including peroxidase (POD), catalase (CAT), superoxide dismutase (SOD), ascorbate peroxidase (APX), and glutathione peroxidase (GPX), increased with decreasing Ψs. In contrast, CAT and POD were higher at 20 °C and 40 °C but declined at 25, 30, and 35 °C. The APX and GPX remained unchanged at 20, 25, 30, and 40 °C but declined at 35 °C. Thus, maintaining enzymatic activity is a protective mechanism against oxidative stress. A decline in germination characteristics may result from energy diverting to anti-stress tools (antioxidant enzymes) necessary for eliminating reactive oxygen species (ROS) to reduce salinity-induced oxidative damage. The parameters examined in this study are easily applicable to simulation models of *Z. mays* L. germination under extreme environmental conditions characterized by water deficits and temperature fluctuations.

## Introduction

Maize (*Zea mays* L.; family Poaceae) is an essential crop species cultivated worldwide. In Pakistan, it ranks third in terms of crop cultivation after wheat and rice. This crop is versatile and is used as fodder, feed, and food source. In addition, it contributes 4% to agricultural value-added and 0.6% to the national gross domestic product [1]. Global food security is under threat due to climate change [2]. In recent decades, environmental constraints such as water stress and temperature have reduced maize production levels, and this decrease is expected to worsen with a changing climate [3]. It is sensitive to abiotic factors which resulted due to these factors, thus including water stress [4, 5] and temperature [6]. The world's annual demand for cereals (maize, rice, and wheat) is expected to surpass 3.3 billion tons by 2050, an increase of 600 million tons from 2019 [7]. To meet continuously rising food demands in a changing climate, adaptation strategies must be developed to maximize the yield of crop species, including maize.

Seed germination (SG) and early seedling growth and establishment are extremely susceptible to environmental factors. For instance, seed germination is a complex physiological process and is highly responsive to external factors such as temperature, water stress and salinity [8–15]. Specifically, SG is sensitive to fluctuating temperature and osmotic potential [16]. A temperature rise can have an adverse effect on the establishment and emergence of plant species due to the relationship among seed germination, temperature, and dormancy. The result of temperature on seed germination is well known [17]. For seed germination, there are three cardinal Ts, namely To (optimal temperature), Tb (low temperature), and Tc (ceiling temperature), which should be understood when determining the appropriate temperature demand and planting date [14, 18]. Moreover, water scarcity is another critical concern for seed germination and juvenile seedlings due to temperature variations, rainfall, and atmospheric humidity fluctuations [19]. There is a direct correlation between germination rate (GR) and variability in water potential surrounding the seedlings. For example, a change in the permeation medium's water potential may slow or halt the germination process [20]. In addition to affecting germination, soil water availability plays a direct and indirect role in all subsequent metabolic processes [21]. Therefore, a water deficit condition during the seed imbibition phase may affect germination percentages (MGT), uniformity, and rates [22, 23]. During the germination process, temperature affects the biochemical reactions. Germination occurs at its peak at the optimal temperature range, although optimal temperatures vary from species to species [21].

In the germination process, mitochondrial respiration increases, producing ROS, which upregulate the antioxidant defence system [24]. The accumulation of ROS, resulting from metabolic changes in response to abiotic stress, leads to oxidative stress, which damages cell membranes (lipid peroxidation), proteins, DNA, and RNA molecules [25–28] and disturbs the normal germination and the establishment of juvenile seedlings. When plants undergo oxidative stress, one of their immediate responses is to eliminate excess ROS via physiological and molecular mechanisms that stimulate enzymes and non-enzymes that remove ROS [25]. Cell damage is affected by the efficiency with which plants activate antioxidant enzymes [29]. Ascorbate peroxidase and catalase are enzymes capable of activating the antioxidant defence system. Therefore, plants that can cope with oxidative stress by upregulating antioxidant enzymes and neutralizing ROS can tolerate stress and continue to grow [30].

For the successful management of crop plants, including maize, several fundamental aspects must be better understood, including their geographic location, behaviour, and physiological metabolism during germination. Using germination models to forecast seed germination under varying temperature ranges and water availability is essential to predicting the behaviour of SG and subsequent seedling emergence.

Researchers [31] have used hydrothermal time (HTT) and hydro-time (HT) models to study SG under different water potentials (ψ) and temperature (T). Using the HTT model, one can determine the concept of fluctuating germination time in response to T ψ and the suboptimal range (from T_b_ to T_o_) and the supra-optimal range (from T_o_ to T_c_) [32]. So far, several plants have adopted this approach, including *Lathyrus sativus* max L. [33], *Carthamus tinctorius* [34]*, Eruca sativa* L. [13] *Triticum aestivum* L*.* [14]*,* and *Trigonella foenum-graecum* L. [12]*.* It is crucial to understand how seeds adapt to drought and high temperatures during seed germination to develop sustainable management approaches for cultivation in the context of future climate change. Moreover, the physiological responses of maize seeds during germination have not yet been identified by scientific parameters. An understanding of how antioxidant enzymes respond to varying temperature and water potential ranges during seed germination can be an important factor in predicting a species' tolerance to environmental stresses in the face of future climate changes*.* Therefore, the purpose of this study was to analyze (a) the effects of varying temperatures and water deficits on germination features, and (b) the responses of antioxidant enzymes during germination. Therefore seed germination, seedling growth, and activities of antioxidant enzymes were assessed.

## Materials and methods

### Petri dish experiment

For the experimental study, at once for the whole experiment seeds of maize (*Z. mays* L.) were obtained from the National Institute of Food and Agriculture (NIFA) in Peshawar, Pakistan. Seeds were sterilized and germinated in the laboratory for the hydrothermal time concept experiment, following a randomized complete block design (RCBD). A Petri dish (with double-layered Whatman No. 1 filter paper) investigation with varying water potentials (*Ψs*; 0 MPa -0.3 MPa, -0.6 MPa, -0.9 MPa, -1.2 MPa; PEG 6000) and temperatures (*Ts*; 20, 25, 30, 35, 40 °C) was conducted in an incubator at the Botany department of the University of Peshawar. There were 15 seeds in each petri dish, having three replicates (a total of 15 treatments were used). Seeds are considered germinated when they reach a length of one millimetre. Several germination characteristics were measured after 96 h after seed germination.

### Agronomic parameters

#### Hydrothermal time model

Based on the HTT concept, sub-optimal and supra-optimal Ts are calculated as follows:

1 $$\mathrm{TTsub}=(\mathrm T-{\mathrm T}_{\mathrm b}),{\mathrm t}_{\mathrm g}\;\mathrm{at}\;\mathrm{sub}-\mathrm{optimal}\;\mathrm T$$

2 $$\mathrm{TTsupra}=({\mathrm T}_{\mathrm c}-\mathrm T){\mathrm t}_{\mathrm g}\;\mathrm{at}\;\mathrm{supra}-\mathrm{optimal}\;\mathrm T$$

TTsub = thermal time at sub-optimal temperature, TTsupra = thermal time at supra-optimal temperature, T = temperature, T_b_ = base temperature, T_c_ = ceiling temperature, t_g_ = thermal time constant.

In the model, each seed percentile 1, (%) is quantified under different thermal time constant [31], proposing the hydro time model (θH) to further improve the model, which explains the relationship between germination rate and solute potential in the same way as the thermal time model does.

3 $${\mathrm{\theta H}}_{\mathrm{g}}= (\uppsi -{\uppsi }_{\mathrm{b}})\mathrm{ tg}$$

#### OR

4 $$\mathrm{Probitg}=\lbrack\mathrm\psi-(\mathrm\theta\mathrm H/t_{\mathrm g})\mathrm\psi-{\mathrm\psi}_{\mathrm b50}\rbrack/{\mathrm\psi}_{\mathrm b}$$

$\uppsi$_b_ = base osmotic potential, $\uppsi$  = average osmotic potential, θH = hydrotime constant,

By combining the HT and TT models, we can calculate and characterize the SG response to various T and Ψ. The HTT may report tg all *T* (from T_b_ to T_o_) and *Ψ*s.

5 $$\mathrm{\theta HTT}= (\uppsi -{\uppsi }_{\mathrm{b}}) (\mathrm{T}-{\mathrm{T}}_{\mathrm{b}}) {\mathrm{t}}_{\mathrm{g}}$$

#### OR

6 $$\mathrm{Probit }(\mathrm{g})=[\uppsi -(\mathrm{\theta htt})/(\mathrm{T}-{\mathrm{T}}_{\mathrm{b}}) {\mathrm{t}}_{\mathrm{g}})-{\uppsi }_{\mathrm{b }50}]/ {\uppsi }_{\mathrm{b}}$$

#### OR

7 $$\mathrm{\theta HTT}= [\uppsi -\uppsi {\mathrm{b}}_{\left(\mathrm{g}\right)} ({\mathrm{k}}_{\mathrm{t}} (\mathrm{T}-\mathrm{To})] (\mathrm{T}-{\mathrm{T}}_{\mathrm{b}}) {\mathrm{t}}_{\mathrm{g}}$$

8 $$\mathrm{Probit}= [\uppsi -{\mathrm{k}}_{\mathrm{t}}(\mathrm{T}-\mathrm{To}) - (\mathrm{\theta H}/(\mathrm{T}-{\mathrm{T}}_{\mathrm{c}})\mathrm{ tg})-{\uppsi }_{\mathrm{b }(50)}]/{\uppsi }_{\mathrm{b}}$$

θHTT = hydrothermal time constant, T = average temperature, T_b_ = base temperature, k_t_ = equation constant, Ψ_b_ = base osmotic potential, Ψ = average osmotic potential,

#### Germination energy (GE)

We calculated the germination energy (GE) using the following formula [35].

9 $$\mathrm{GE }=\frac{X1}{Y1}+\left(\frac{X2-X1}{Y2}\right)+\left(\frac{Xn-Xn-1}{Yn}\right)$$

The terms X1, X2, and Xn in the formula above show the number of seeds that germinated on the first day, second day, and so forth. While Y1, Y2, and Yn stand for the total number of seeds in each Petri dish.

#### Mean germination time (MGT)

MGT is a measure of how quickly seeds germinate in a population. A small MGT will result in a high seed population rate and vice versa. Following is the equation used to calculate the MGT [36].

10 $$\mathrm{MGT}=\frac{\in\mathrm{fx}}{\in\mathrm f}$$

F = germinated seeds, x = time(days), f = total number of seed germinated.

#### Germination Index (GI)

The GI represents the percentage of seeds that have germinated at a specific time of the day [37].

11 $$\mathrm{GI}=(10\times n1)+(9n\times n2)+...\,...\,...\,...+(1n\times n10)$$

The No. of seeds that germinated on days 1, 2, and 10 were indicated by the letters n1, n2,…, and n10. While 10, 9, and 1 reflect the weighted average of seeds number that germinated on days.

#### Mean Germination Rate (MGR)

Using the following equation [37], we calculated the MGR.

12 $$\mathrm{MGR} =\frac{1}{\mathrm{MGT}}$$

#### Coefficient of Velocity of Germination (CVG)

The seed germinating rate is reflected in CVG and increases with the increased frequency of germinated seeds. Following is the formula used to calculate CVG [37].

13 $$\mathrm{CVG }=\frac{N1+\mathrm{N}2+\mathrm{N}3\dots \mathrm{Nx}}{100}\times \mathrm{N}1\mathrm{T}1\dots .\mathrm{NxTx}$$

N = number of seeds, T = corresponding days.

#### Germination Rate Index (GRI)

GRI was calculated, based on the following equation [38].

14 $$\mathrm{GRI}=\frac{\mathrm{G}1}{1}+\frac{\mathrm{G}2}{2}+\frac{\mathrm{G}3}{3}\dots .\frac{\mathrm{Gx}}{\mathrm{x}}$$

G1 and G2 represent the percentage of germinating seeds one day 1, 2 respectively, and so on.

### Determination of antioxidant enzymes activities

We ground and homogenized fresh leaf samples in phosphate buffer (0.1 M; pH 7.3) containing EDTA (0.6 mM). We centrifuged the mixture at 12,000 × *g* (10 min; 4 °C). Next, the enzyme extract contained in the supernatant was then used for the assays.

#### Estimation of catalase (CAT) activity

The CAT activity was assessed by monitoring the disappearance of H_2_O_2_ [39]. The enzyme extract was added with a reaction mixture of K-phosphate buffer (50 mM; 7.0) and hydrogen peroxide (15 mM). The absorbance was read at 240 nm using a spectrophotometer.

#### Peroxidase (POD) activity

The activity of POD was determined by standard methods [40]. We mixed enzyme extract (0.5 ml) with buffer substrate (guaiacol and Na_3_PO_4_ pH 6.4), and H_2_O_2_ (24 mM). The absorbance at 460 nm was measured twice at intervals of one minute.

#### Superoxide dismutase (SOD) activity

Based on a standard method, we measured the superoxide dismutase (SOD) activity [41]. A reaction mixture containing phosphate buffer (50 mM; pH 7.8), riboflavin (2 mM), nitroblue tetrazolium (75 mM), and methionine (130 mM) contained approximately 0.1 ml of enzyme extract. Using a spectrophotometer, we determined SOD based on the decline rate of nitroblue tetrazolium at 560 nm.

#### Ascorbate peroxidase (APX) activity

The APX activity was measured following a standard method [42]. The reaction mixture was prepared as follows: Phosphate buffer (50 mM; pH 7.0), hydrogen peroxide (1 mM), L-ascorbic acid (0.25 mM), and enzyme extract (0.1 ml). With a spectrophotometer, a rise in absorption (290 nm) was noted following ascorbate oxidation.

#### Guaiacol peroxidase (GPX) activity

Guaiacol peroxidase was measured according to [43]. The enzyme assays were prepared by adding 0.5 ml 0.1 M K-phosphate buffer (7.5), 0.5 ml 3.4 mM guaiacol (0.5 ml), H_2_O_2_ (0.5 ml), and enzyme extract (0.5 ml) to a glass cuvette. The absorbance at 480 nm was measured by measuring the amount of guaiacol oxidized.

### Statistical analysis

Statistical computations were carried out using Excel software. In SPSS statistic 25, the linear probit regression analysis was used to determine the values of the variables: σΨ_b_, Ψ_b_ (50), R^2^, SE, F, T-test, and Sig. Germination fractions and germination parameters were compared using Origin 2021 PC Corporation Graphs of germination parameters against Ψ, and T were created using Origin 2021 PC Corporation.

## Results

### Germination response to temperature and osmotic potential

**Fig. 1:**
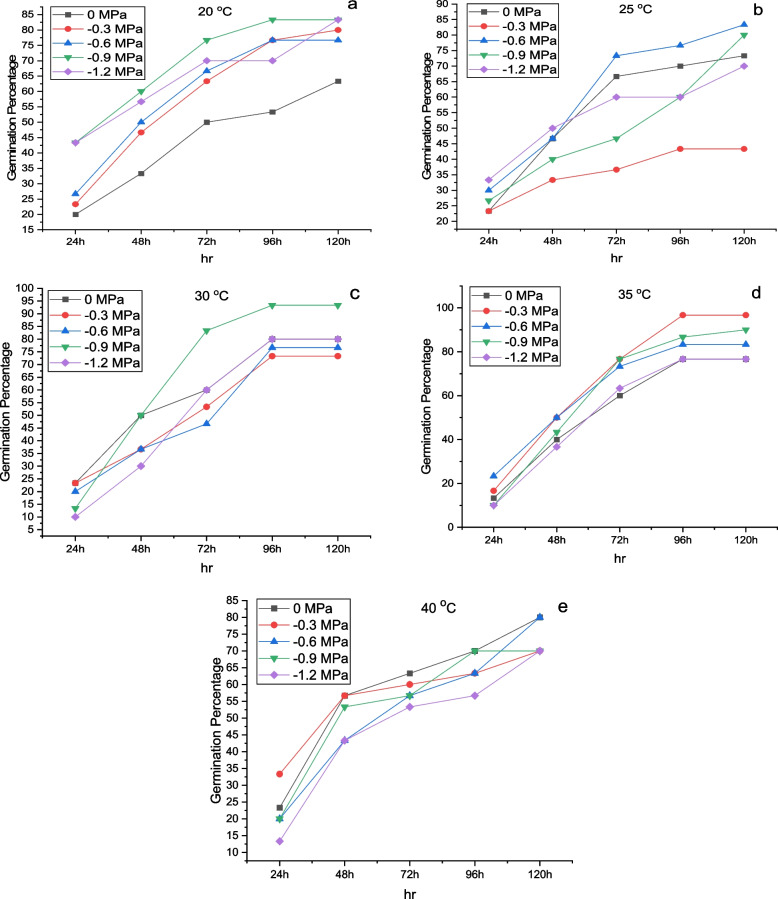
Changes in cumulative germination for maize at (**a**) 15 °C (**b**) 20 °C (**c**) 25 °C (**d**) 30 °C (**e**) 35 °C and (**f**) 40 °C with different ψ. The symbols indicate the water potential and the lines indicate the cumulative germination rate

**Fig. 2:**
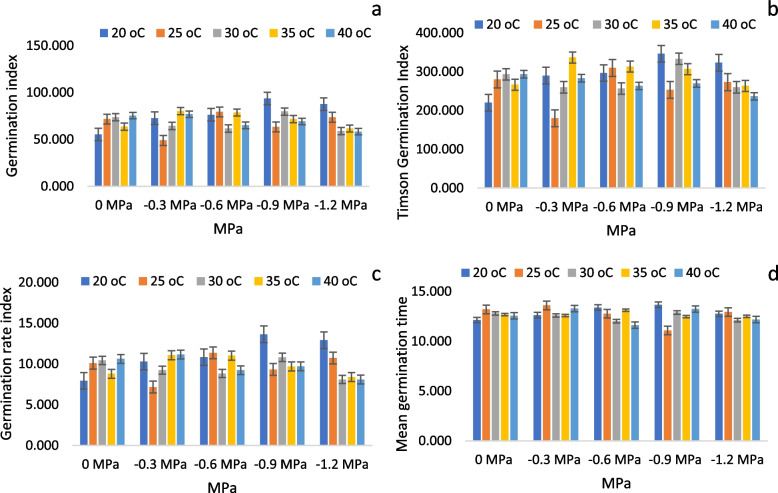
Effects of varying T and ψ on (**a**) germination index (**b**) Timson Germination Index (**c**) germination rate index and (**d**) mean germination time of maize using HTT

As temperature amplitude increased, germination rates and percentages increased, but this decreased once T reached a particular threshold. As shown in Fig. 1a-e, the GP was highest at 35 °C (-0.3 MPa) and lowest at 25 °C (-1.2 MPa). The minimum values of GP, 10%, and 13.33% were found to be recorded at 20 °C under (-1.2 MPa), while the maximum value of 100% was recorded at 35 °C under (-0.3 MPa). There was a maximum GI (germination index) and TGI (timson germination index) at 20 °C, at (-0.9 MPa) while the minimum GI and TGI at 25 °C, at (-0.3 MPa). Furthermore, the mean germination time (MGT) was highest at 25 °C (-0.3 MPa) and lowest at 25 °C (-0.9 MPa), while the germination rate index was highest at 20 °C (-0.9 MPa) (Fig. 2a-d). There was maximum germination energy (GE) at a temperature of 20 °C in (-0.9 MPa) and a minimum at a temperature of 30 °C in (-1.2 MPa).

**Fig. 3:**
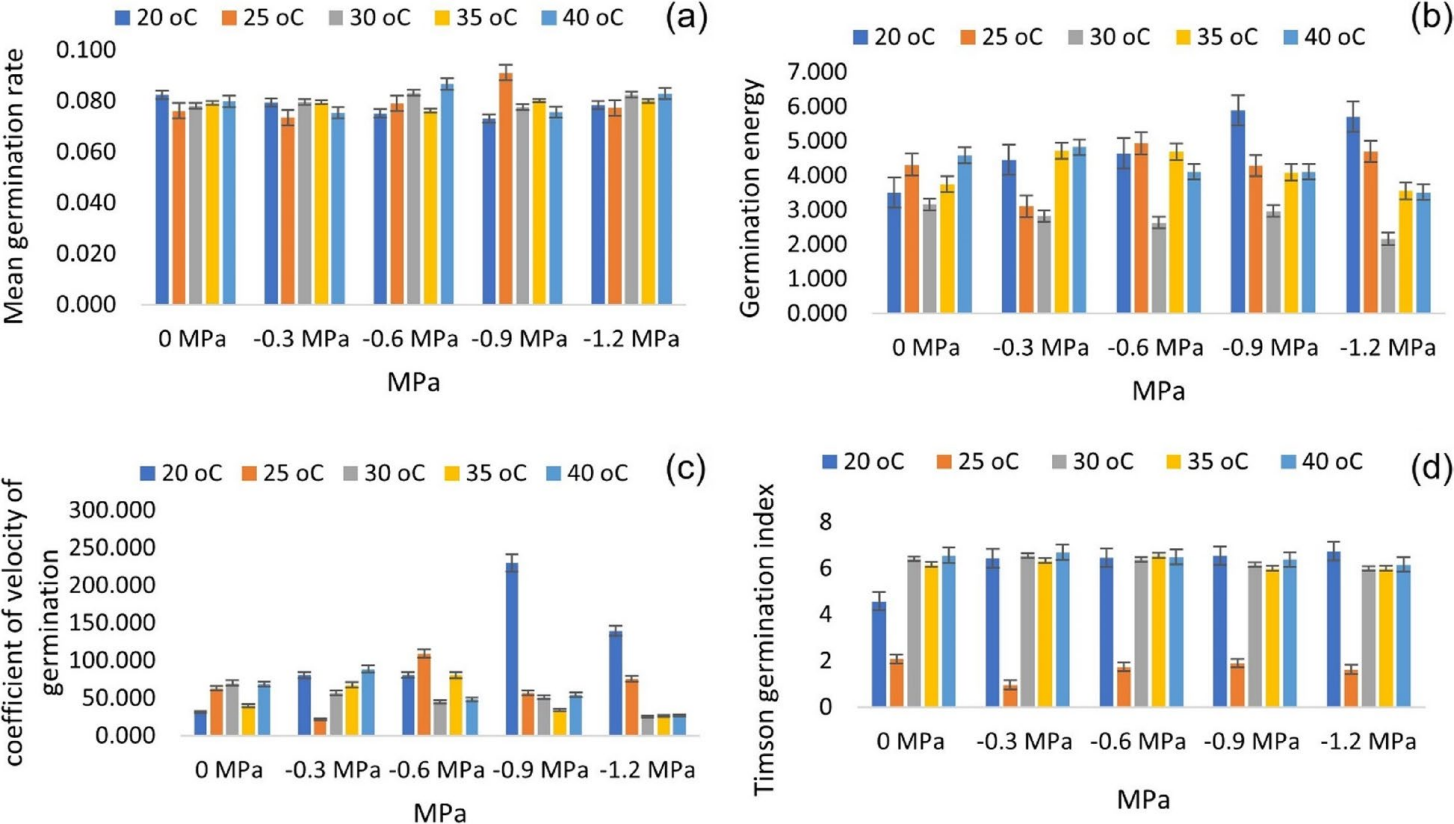
Effects of varying T and ψ on (**a**) mean germination rate (**b**) germination energy, (**c**) the coefficient of germination velocity and (**d**) Timson germination index of maize based on the HTT model

Conversely, the mean germination rate (MGR) was lowest at 20 °C (-0.9 MPa) and highest at 25 °C (-0.9 MPa). Furthermore, the highest value of CVG was recorded for 20 °C, at -0.9 MPa, while the lowest value of CVG was recorded for 20 °C, at -0.3 MPa (Fig. 3a-d). Based on the results of the HTT experiment, it was predicted that the temperature and water potential significantly affected the germination parameters.

We noticed the maximum θT1 value at 40 °C, at 0 MPa, and the minimum at 20 °C (0 MPa). In contrast, the highest value of θT2 was reported at -0.9 MPa at 20˚C and minimum at 40 ˚C (-1.2 MPa) (Table 1). It has been found that the TT theory is well suited to germination data in distilled water, with R^2^ increasing by 0.43. Seed germination can be investigated using the hydrothermal time model by considering the influence of water potential and temperature above thermal and hydro thresholds. It has a higher value (R^2^ = 0.43 at 30 °C) at suboptimal temperatures (T < T0) than at supra-optimal temperatures (R^2^ = 0.41). Our experiment used 20 °C as the base temperature (Tb). Below this temperature, the seed of maize growth is prolonged, and a plant will have difficulty maintaining physiological functions. Plant growth decreased above the optimum temperature, with the lowest growth observed at 40 °C (Table 1).

**Table 1:** The hydro and thermal time models' estimated parameters describe *Z. mays* L. seed germination under fluctuating different temperatures (Ts) and water potentials (ψs)

Temperature	treatment	TTsub/θT1	TTsupra/θT2	θH(MPa h)	θHTT (MPa h)	TT GR	HT GR
20	0 MPa	184.00	1104.00	55.20	276.00	0.028	0.028
	-0.3 MPa	243.20	1459.20	73.26	291.84	0.022	0.017
	-0.6 MPa	244.00	1464.00	72.60	219.60	0.022	0.013
	-0.9 MPa	274.40	1646.40	81.42	164.64	0.020	0.008
	-1.2 MPa	255.20	1531.20	76.16	76.56	0.020	0.004
25	0 MPa	462.40	1156.00	69.36	693.60	0.022	0.022
	-0.3 MPa	283.20	708.00	42.48	339.84	0.036	0.029
	-0.6 MPa	512.00	1280.00	76.80	460.80	0.020	0.012
	-0.9 MPa	425.60	1064.00	63.84	255.36	0.024	0.009
	-1.2 MPa	433.60	1084.00	65.04	130.08	0.023	0.005
30	0 MPa	736.80	982.40	73.68	1105.20	0.020	0.020
	-0.3 MPa	660.00	880.00	66.00	792.00	0.023	0.018
	-0.6 MPa	664.80	886.40	66.48	598.32	0.023	0.014
	-0.9 MPa	866.40	1155.20	86.64	519.84	0.017	0.007
	-1.2 MPa	698.40	931.20	69.84	209.52	0.022	0.004
35	0 MPa	924.80	693.60	69.36	1387.20	0.023	0.023
	-0.3 MPa	1168.00	876.00	87.60	1401.60	0.017	0.014
	-0.6 MPa	1049.60	787.20	78.72	944.64	0.019	0.012
	-0.9 MPa	1078.40	808.80	80.88	647.04	0.019	0.007
	-1.2 MPa	924.80	693.60	69.36	277.44	0.022	0.004
40	0 MPa	1194.00	477.60	71.64	1791.00	0.022	0.022
	-0.3 MPa	1103.33	441.33	66.20	1324.00	0.023	0.018
	-0.6 MPa	1103.33	441.33	66.20	993.00	0.023	0.014
	-0.9 MPa	1098.00	439.20	65.88	658.80	0.023	0.009
	-1.2 MPa	992.67	397.07	59.56	297.80	0.026	0.005

### Physiological response to temperature and osmotic potential

The antioxidant enzymes were significantly affected by fluctuations in temperature and osmotic potential. We obtained the highest significant values of CAT, POD, APX, and GPX at 20 °C at -1.2 MPa. It was found that the highest value of SOD was recorded at 40 °C in -1.2 MPa while the lowest value was recorded at 25 °C in control. The lowest values of CAT, POD, APX and GPX were measured at 35 °C in the control group. Observations have shown that all enzymes respond normally to (0 MPa) temperatures between 25 and 30 °C. At both the highest and lowest treated temperatures, however, the effect was adverse. When comparing all the osmotic and thermal responses, it was antioxidant enzymes that had the most remarkable response at 20 °C (-1.2 MPa).

**Fig. 4:**
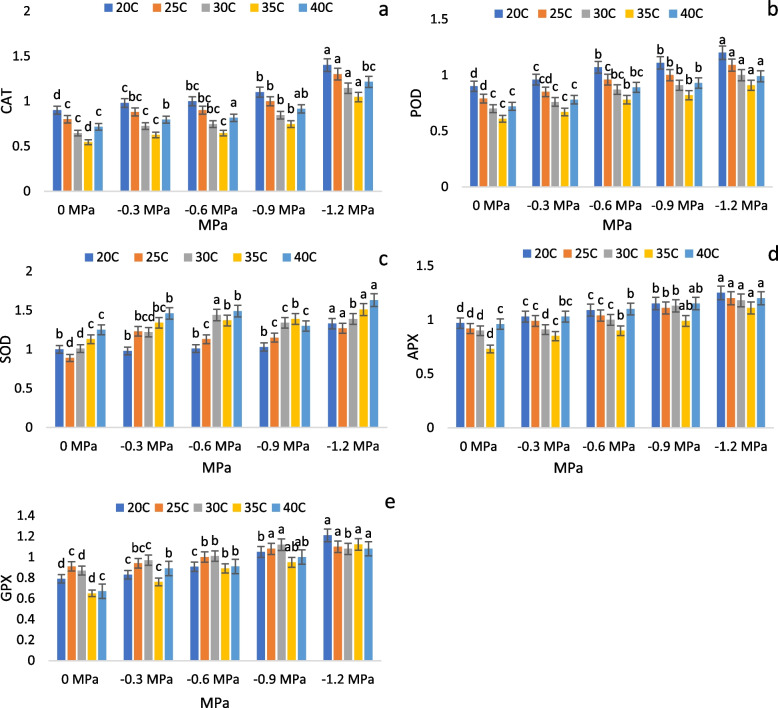
Effects of varying T and ψ on (**a**) CAT (**b**) POD (**c**) SOD (**d**) APX and (**e**) GPX activities in maize seedlings

Furthermore, all antioxidant enzymes showed the lowest response at 0 MPa (Fig. 4).

### Correlation, Heat map and PCA results of Germination and antioxidants enzymes to fluctuating temperatures and water potentials

**Fig. 5:**
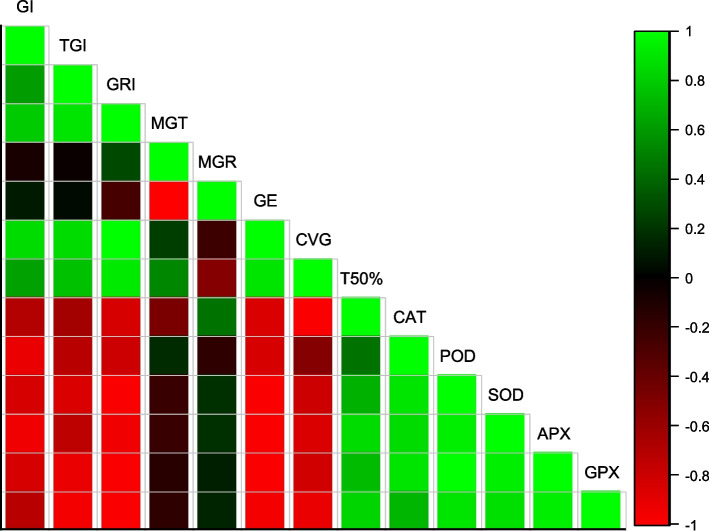
Correlation of germination features and activities of antioxidant enzymes in maize seedlings under varying T and ψ

**Fig. 6:**
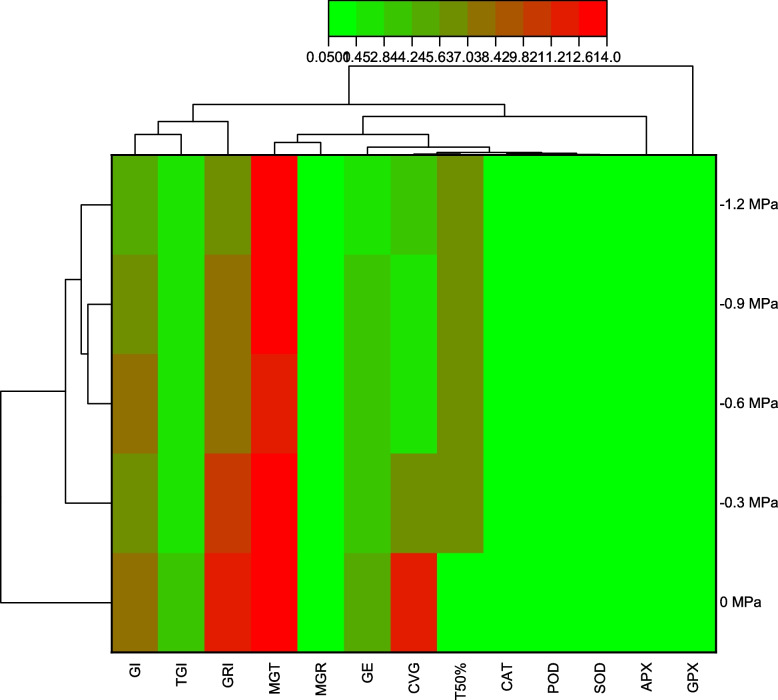
Heatmap correlation of germination features and activities of antioxidant enzymes in maize seedlings under varying T and ψ

**Fig. 7:**
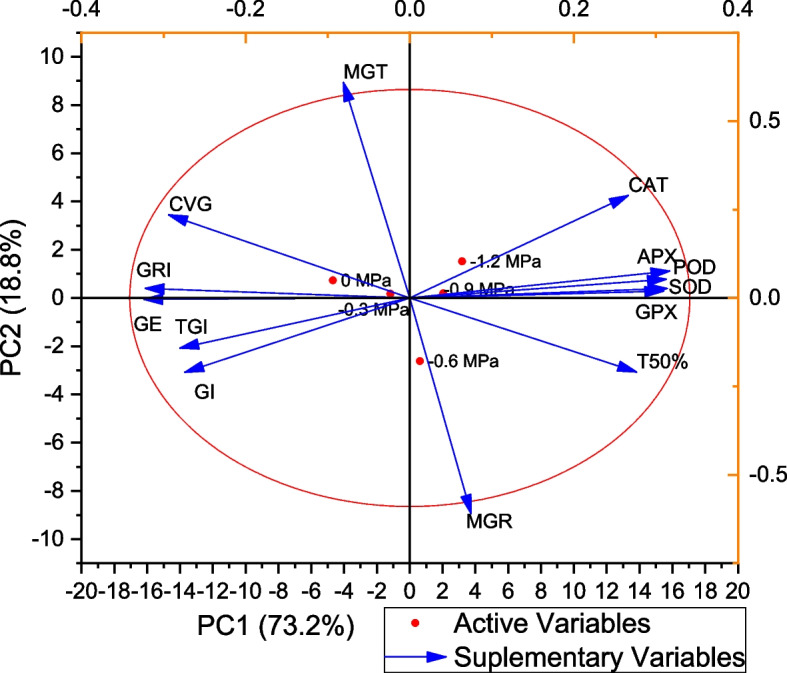
Loading Plot of Principal component analysis (PCA) on germination features and activities of antioxidant enzymes in maize seedlings under varying T and ψ

GI was positively correlated with TGI, GRI, MGT, CVG, and T50%, and negatively correlated with CAT, SOD, POD, APX, and GPX. Antioxidant enzymes are positively correlated with each other and negatively correlated with all aspects of germination (Fig. 5). In all parameters, the heatmap histogram shows two distinct clusters. The first cluster consists of 0 MPa, while the second cluster consists of -0.3, -0.6, -0.9, and -1.2 MPa (Fig. 6). We used PCA for the analysis of enzymes and germination parameters. Based on the results of PCA, all treatments are distributed evenly throughout the dataset except for SOD and POD. The parameters distribution shows that the Ψ had a high impact on the germination. Using a PCA base biplot, the findings revealed that the first two components accounted for 73.2% of the overall variation since the first two components were the most variable (Fig. 7).

## Discussion

The optimal geographic location of a species could be determined by evaluating its germination patterns and underlying physiological mechanisms under various environmental conditions. Seed germination can be quantified using mathematical models (TT, HT, and HTT). The prediction of maize germination using multiple germination models and modulation of physiological responses can provide insight into adoption mechanisms and agronomic management programs in response to constantly changing environmental conditions. The HT, TT, and HTT models evaluate the impact of diverse abiotic variables on seed germination time in various seed lots [13]. T and ѱ are highly significant environmental variables affecting seed germination in many plants [13, 15]. Furthermore, our experiment suggested that increasing T and ѱ had a substantial impact on SG. The temperature response of seeds can be characterized by their cardinal temperatures (i.e., T_b_, T_o_, and T_c_). In this study, the hydrothermal time model (HTT) was used to determine the germination features and antioxidant mechanism of maize seedlings under different ranges of water potential (Ψs; 0. -0.3, -0.6, -0.9, -1.2 MPa) and temperature (Ts; 20, 25, 30, 35, 40 °C). The temperature plays an important role in the germination process of seeds. We observed that the cardinal temperatures such as base temperature (Tb, at which germination decreases), optimal temperature (To, at which germination is optimal), and ceiling temperature (Tc, above which germination is halted) were recorded as 20 °C, 30 °C, and 40 °C, respectively (Table 2). The germination percentage (GP) was highest at 35 °C and 0 MPa, while germination was lowest at 20 °C and 0 MPa. The Cardinal temperatures for germination depend on the range of environmental adaptation of a particular species. As a result, they ensure that the germination time and favourable conditions are matched so that seedlings can grow and develop successfully [18]. It has been demonstrated that germination rates increase between minimum and optimum temperatures, while germination rates decrease between optimum and maximum temperatures [44]. The germination process is likely to need to adapt to certain changes in environmental conditions as a result of global climate change, among other factors. It has been suggested by Gummerson [34] that GP decreases as a result of the denaturation of critical amino acids and an alteration in physiological responses to high temperatures. In our study, the maximum GP was observed at 30 °C, compared with the control. Temperature appears to influence many plant species' GP and GR, which is a critical factor in seed germination. Thus, species whose optimum temperatures can be extended to both ends (minimum and maximum temperature ranges) to maintain their germination characteristics and physiological responses of anti-stress mechanisms will have advantages to ensure their seedling's establishment under continuously changing climatic conditions, including temperature fluctuations. Another factor that influences seed germination is water potential (Ψ). Additionally, we recorded in our experiment that water potential (Ψ) greatly impacted seed germination. A possible reason for this could be that these seeds were dried from a fully hydrated state, so they could not complete the germination process [44]. Similar reported for watermelon, potatoes [45], zucchini [8], watermelon [13], and wheat [14].

**Table 2:** Estimated germination and cardinal temperature values for *Z. mays* L. using the hydrothermal time model

**Hydrothermal time model parameters**	
Variables	
*Ѱ*_b_ (50) (MPa)	-1.13
σ*ψ*_b_ (MPa)	0.21
θH (MPa˚Ch^−1^)	70.17
k_T_ (MPa˚Ch^−1^)	0.104
**Cardinal temperatures**	
*T*_b_ (˚C)	20
*T*_o_ (˚C)	30
*T*c (˚C)	40
*R*^*2*^	0.43

Our findings indicate that for all cardinal temperatures Ts, the GR increased (*p* ≤ 0.01) as the osmotic potential decreased (more negative) (Table 1). When Ψ was lowered in comparison to the control, GR decreased. We observed a minimum temperature of 20 °C in our experiment (Tb). The optimal temperature (To) for plant germination growth was 30 °C, and the maximum temperature (Tc) for physio-biochemical activity was 40 °C. As described by [12], the germination of seeds has three cardinal temperatures (Ts) that are critical in defining the characteristics of SG.

Finally, we observed that the HTT model could be used to describe how environmental factors (Ψ and T) influence seed germination in seed lots. Following Table 2, the hydro-time constant (HT) calculated for *Z. mays* was 70.17 (MPa Ch^−1^). Compared with high T and low T, agronomic germination parameters such as GP, TGI, GRI, GE and GI were reduced. Our studies align with previous studies on different plant species [13, 14, 46, 47]. This occurs due to the seed's thermo-inhibiting effects on cellular and chemical processes. Statistical analysis confirms the impact of cardinal temperatures and HTT on the SG population by explaining their interactions. Furthermore, soil water availability and temperature variation affect all subsequent metabolic processes and germination [21, 48, 49]. The highest (87.60) and lowest (55.20) hydro-time constant (θH) were reported at 35 °C and 20 °C, respectively. The highest and lowest base water potential (50 percentiles) (-1.07 and -1.02 MPa, respectively) were reported at 30 °C and 20 °C. Furthermore, the Tc, To, and Tb were determined as 40 °C, 30 °C, and 20 °C, respectively. HTT has a higher value (R^2^ = 0.43 at 40 °C) at sub-optimal than supra-optimal temperatures (R^2^ = 0.41 at 40 °C).

Furthermore, it is known that plants are sensitive to water and temperature changes in their environment. They can trigger specific pathways of biochemical and molecular responses [48]. A high temperature during the incubation of seeds, for instance, induces oxidative stress, which results in increased production of reactive oxygen species (ROS) and subsequent metabolic events within the cell [49]. Furthermore, when seeds are rehydrated following imbibition, the physiological responses are triggered, allowing germination to take place [50]. Therefore, it is important to study germination characteristics in conjunction with physiological mechanisms to gain a deeper understanding of the response and adaptation to environmental variables that affect seed germination and establishment of seedlings. The ROS must be controlled by antioxidant systems at low levels during germination to prevent cell damage and disruption of metabolic processes [51]. There have been numerous consequences associated with the intoxication of seeds by ROS. These include a reduction in ATP production [49], lipid peroxidation, rupture of the cell membrane [52], biomolecular alterations and loss of seeds viability [51]. In this study, we observed that CAT, POD, SOD, APX, and GPX increased with decreasing Ψ. The CAT and POD were higher under 20 °C and decreased with rising temperatures (25-, 30-, and 35 °C). Interestingly their activities increased at 40 °C (> 25-, 30, and 35 °C). Furthermore, SOD also increased with increasing Ts. In addition, APX and GPX remained unchanged at 20-, 25-, 30-, and 40 °C but decreased at 35 °C. It has been suggested that germination increases mitochondrial respiration, which produces excess reactive oxygen species (ROS) that activate antioxidant defences [24] to protect themselves from ROS-induced oxidative damage [53–57], which corroborates our findings. CAT, POD, GPX, and APX are the major ROS-scavenging enzymes that reduce H_2_O_2_ to reduce potential cellular damage associated with H_2_O_2_. The maintenance of enzymatic activity is a protective mechanism against oxidative stress damage. The decline in germination characteristics might be due to energy being diverted to anti-stress tools (antioxidant enzymes) essential for eliminating ROS generated during mitochondrial respiration at the time of germination. Such kinds of studies may assist in deducing the optimal temperature and water potential for the germination of crop species, as well as the adaptive response mechanisms at the juvenile stage of a plant's development, which is the most sensitive stage. However, the complex physio-biochemical and molecular responses of the seed populations of the test species to abiotic factors should be accounted for in the model's parameters for predicting future germination times.

## Conclusions

The increasing Ψs and Ts significantly impacted the germination attributes. The highest (87.60) and lowest (55.20) hydro-time constant (θH) were reported at 35 °C and 20 °C, respectively. The highest and lowest base water potential (50 percentiles) -1.23 and -1.02 MPa, respectively) were reported at 35 °C and 25 °C (Table 3). Furthermore, the Tc, To, and Tb were determined as 40 °C, 30 °C, and 20 °C, respectively. HTT has a higher value (R^2^ = 0.43 at 40 °C) at sub-optimal than supra-optimal temperatures (R^2^ = 0.41 at 40 °C). The maintenance of enzymatic activity is a protective mechanism against oxidative stress damage. Germination characteristics may decline due to energy being diverted to anti-stress tools (antioxidant enzymes) essential for eliminating ROS generated during mitochondrial respiration at the time of germination. Such studies may be useful in determining the optimal temperature and water potential for germination of crop species, as well as the adaptive mechanisms at the juvenile stage, the most sensitive phase of the plant's development. Due to future climate change and rising food demands, the prediction of germination models along with adoptive physiological response mechanisms could provide insight into the ideal conditions for producing optimal germination, physiological adjustments, growth and productivity. However, the complex physio-biochemical and molecular responses of the seed populations of the test species to abiotic factors should be accounted for in the model's parameters for predicting future germination times.

**Table 3:** Estimation of hydro-time model parameters for *Z. mays* L. using non-linear regression

Temperature	*ѱ*_b_(_50)_ (MPa)	σ*ψ*_b_ (MPa)	*R*^*2*^	SE	F	T	Sig
20˚C	-1.12	0.27	0.14	1.11	2.32	6.91	0.14
25˚C	-1.02	0.24	0.27	1.25	1.14	21.20	0.82
30˚C	-1.18	0.17	0.18	1.33	1.42	20.84	0.86
35˚C	-1.23	0.20	0.43	1.42	7.08	1.43	0.11
40˚C	-1.07	0.18	0.41	1.74	6.97	18.58	0.14

## Data Availability

Not applicable.
